# Environmental stability of porcine respiratory coronavirus in aquatic environments

**DOI:** 10.1371/journal.pone.0254540

**Published:** 2021-07-14

**Authors:** Maarten De Rijcke, Hisham Mohammed Shaikh, Jan Mees, Hans Nauwynck, Michiel Bert Vandegehuchte

**Affiliations:** 1 Flanders Marine Institute (VLIZ), InnovOcean Site, Oostende, Belgium; 2 Marine Biology Research Group, Ghent University, Faculty of Sciences, Ghent, Belgium; 3 Laboratory of Virology, Faculty of Veterinary Medicine, Ghent University, Merelbeke, Belgium; Michigan Technological University, UNITED STATES

## Abstract

Coronaviruses (CoVs) are a family of viruses that are best known as the causative agents of human diseases like the common cold, Middle East Respiratory Syndrome (MERS), Severe Acute Respiratory Syndrome (SARS) and COVID-19. CoVs spread by human-to-human transmission via droplets or direct contact. There is, however, concern about potential waterborne transmission of SARS-CoV-2, the virus responsible for COVID-19, as it has been found in wastewater facilities and rivers. To date, little is known about the stability of SARS-CoV-2 or any other free coronavirus in aquatic environments. The inactivation of terrestrial CoVs in seawater is rarely studied. Here, we use a porcine respiratory coronavirus (PRCV) that is commonly found in animal husbandry as a surrogate to study the stability of CoVs in natural water. A series of experiments were conducted in which PRCV (strain 91V44) was added to filtered and unfiltered fresh- and saltwater taken from the river Scheldt and the North Sea. Virus titres were then measured by TCID_50_-assays using swine testicle cell cultures after various incubation times. The results show that viral inactivation of PRCV in filtered seawater can be rapid, with an observed 99% decline in the viral load after just two days, which may depend on temperature and the total suspended matter concentration. PRCV degraded much slower in filtered water from the river Scheldt, taking over 15 days to decline by 99%, which was somewhat faster than the PBS control treatment (T_99_ = 19.2 days). Overall, the results suggest that terrestrial CoVs are not likely to accumulate in marine environments. Studies into potential interactions with exudates (proteases, nucleases) from the microbial food web are, however, recommended.

## 1. Introduction

The emergence and rapid spread of COVID-19, a severe respiratory disease caused by the newly discovered single stranded RNA coronavirus SARS-CoV-2, has affected public health, economy, and society on a global scale. SARS-CoV-2 and other human coronaviruses (HCoV) are primarily transmitted from person to person through the direct inhalation of droplets produced by coughing, sneezing or breathing, but other routes like hand-mediated contact and fecal-oral transmission have also been described [1, 2]. Even though SARS-CoV-2 primarily causes infection of the upper respiratory tract, SARS-CoV-2 RNA is found in the faeces of both symptomatic and asymptomatic patients [3]. First reports at the start of the pandemic suggested that patients shed SARS-CoV-2 via their faeces for extended periods [4, 5]. These findings were quickly followed by the detection of SARS-CoV-2 in wastewater [6–8] as well as rivers [9–11] while concurrent in vitro studies were showing that SARS-CoV-2 remains active for up to 25 days at 5°C in aquatic environments [12] and can survive in water with the pH values ranging from 3 to 10 [13]. These early reports led to concerns that SARS-CoV-2 might persist and accumulate in coastal seawater. To date, there is little data on the stability of coronaviruses in saltwater environments. Around the summer of 2020 this knowledge gap became crucial as local or regional governments were trying to assess the risk of SARS-CoV-2 infection during recreational beach use.

Aquatic coronaviruses are present in the marine environment but they are generally uncommon, and uniquely marine betacoronaviruses–the genus that contains human pathogens like MERS, SARS-CoV and SARS-CoV-2—have not yet been discovered [14]. Terrestrial coronaviruses are introduced in coastal waters by sewage and river effluents, but the virions are expected to rapidly degenerate in surface waters [14]. Marine phages and eukaryotic viruses typically decay at rates of 2–4% h^-1^ [15–17], which is thought to be caused by nucleases and proteases of microorganisms [18] as well as exposure to UV-C radiation [19]. Marine viruses have different particle decay rates and species-specific loss of infectivity [19, 20]. Species-specific decay due to these mechanisms is also expected for terrestrial viruses entering the marine environment. Some pathogenic enteric viruses (e.g. enteroviruses, hepatitis A viruses, Norwalk viruses, adenoviruses, rotaviruses, etc.) are notoriously able to maintain infectivity in the marine environment. The stability of these non-enveloped viruses in seawater has been studied extensively [21]. By contrast, little is known about the stability of free coronaviruses (CoVs) in aquatic environments.

To date, only a handful of studies have examined the persistence free CoVs in water and virtually all available studies have used freshwater media (reagent-grade, tap water, lake water, sewage) [22, 23]. Both human and surrogate animal CoVs are used in these studies. Overall, the results of these studies suggest that CoVs are generally unstable in aquatic environments, but more studies on the inactivation rates are needed [22]. In the present study we investigate the persistence of porcine respiratory coronavirus–a new surrogate organism for free CoVs—in fresh- and saltwater environments to acquire additional data on the stability of CoVs in natural aquatic ecosystems. To this end, three consecutive experiments were performed using water from the river Scheldt and the North Sea as natural freshwater and saltwater matrices.

## 2. Material and methods

### 2.1. Pilot studies

One litre of natural water was taken from the river Scheldt (51° 2’ 33.9648’’ N, 3° 44’ 28.896’’ E) and the North Sea (51° 14’ 23.5428’’ N, 2° 55’ 40.7568’’ E) on June 5^th^, 2020. Sediment, plankton and other debris were removed using a 150 μm mesh filter followed by subsequent filtrations over 10 μm and 0.2 μm Whatman PTFE membrane filters (VWR, Leuven, Belgium). The salinity of the filtrates was then measured with a HI-96822 refractometer (Hanna Instruments, Bedforshire, UK). Artificial seawater was prepared in accordance to the guidelines of the American Society for Testing and Materials [24], while a common bottle of mineral water (Chaudfontaine^tm^) was used as an artificial freshwater treatment. Porcine respiratory coronavirus PRCV strain 91V44 [25] was grown on swine testis (ST) cells and added 1 mL aliquots of the four media at the third passage on confluent monolayers of ST cells. Phosphate buffered saline (PBS) was used as a positive control, while all media were used without PRCV as negative controls. Half of the aliquots were incubated at 15°C, the natural water temperature at the time of the experiment, the other half were incubated at 20°C (i.e. average summer water temperature). No replicates were included in the design, but the experiment was performed twice. The first experiment ran for 4 hours, with titres being determined at 0, 2 and 4 hours. The second experiment ran for 3 days, with titrations being performed after 0, 1 and 3 days.

PRCV titres were determined using a Tissue Culture Infectious Dose 50%/mL (TCID_50_) assay with confluent monolayers of ST cells. Samples of the different media were first made isotonic to the ST cells (9 PSU) before the start of TCID_50_ assay to avoid osmotic shock. Freshwater samples (500 μl; 0 PSU) were mixed with 45 μl of 10x PBS (Sigma-Aldrich, Steinheim, Germany), whereas 1.2mL deionized water was added to seawater samples (32 PSU). The dilutions were accounted for in the calculation of the PRCV titres. PBS samples were not changed. Tenfold dilutions were then made by adding 900 μl ST medium (Minimal Essential Medium supplemented with 10% Fetal Calf Serum, 100 U/mL penicillin and 0.1 mg/mL streptomycin) to 100 μL of sample. 50 μL of each dilution was added to four wells of a 96 well tissue culture plate containing confluent monolayers of ST cells. Inoculated plates were incubated at 37°C. After 1 hour an additional 100 μl of ST medium was added to each well. Plates were inspected after 24 hours to ensure that no osmotic shock had occurred. After four days of incubation the monolayers were inspected for cytopathic effects using light microscopy. Virus titres were calculated using the Reed and Muench method [26]. The time required for virus titres to decrease 50%, 90%, 99%, 99.9% and 99.99% (T_50_, T_90_, T_99_, T_99.9_ & T_99.99_) was estimated using the slope of the linear regression.

### 2.2. Suspended particles

To explore potential interactions between PRCV and suspended particles (both biotic and abiotic), another short experiment was performed. Natural seawater was taken from the North Sea on the 9^th^ of August 2020. A 150-μm mesh filter was used to remove larger sized debris as well as any zooplankton that could feed on the phytoplankton and other organic particles. In addition to using the coarsely filtered seawater, we also increased the concentration of suspended particles using tangential flow ultrafiltration to enhance the likelihood of interactions. To this end, 5L of seawater was concentrated to 500 mL and then on to 50 mL using Vivaflow 50 100 kDa crossflow cassettes (Sartorius, Goettingen, Germany) connected to a Cole-Parmer Masterflex L/S peristaltic pump (VWR, Leuven, Belgium). This procedure resulted in three saltwater media containing 1x, 10x, or 100x the natural concentration of suspended particles (> 100 kDa). Reagent-grade PBS was again used as a positive control. PRCV from the same stock and strain as before was added to 10 mL aliquots of each of the four treatments (i.e., PBS; 1x; 10x; 100x natural seawater). The aliquots were then incubated at 20° C on a low-speed rocker shaker to prevent sedimentation. Because of the risk of microbial growth in the seawater treatments, we opted for a short incubation of 6 hours. At the start and end of the experiment (0 and 6 hours) a 1 mL sample was taken to determine the PRCV titres using the same TCID_50_ assay. To prevent bacterial growth on the tissue cultures, an additional filtration step was required to remove all suspended particles. To this end, syringe filters of 0.2 μm and 0.45 μm (Sarstedt, Nümbrecht, Germany) were used. The filtrates were treated as before: we diluted the samples to isotonic conditions and added them to ST cell cultures to determine the PRCV titres using the same TCID_50_ assay.

### 2.3 Long-term stability

After these initial experiments, a longer-term experiment was set up. On the 2^nd^ of October 2020, water was again collected from the North Sea and the river Scheldt and prepared as described for the pilot studies. This time the artificial treatments (ASTM & Chaudfontaine) were not used. Instead, the experiment was performed in triplicate. A 30 mL stock solution was prepared for each medium by adding 0.6 mL of viral stock (PRCV strain 91V44) to 29.4 mL of PBS, filtered seawater, or filtered river water. Each stock solution was briefly vortexed before splitting the solution in three equal 10 mL replicates. All media were incubated at 20° C for 49 days. 500 μL subsamples were taken every 7 days to determine the PRCV titres as before, but now 6 wells were used per dilution instead of four to increase the accuracy of the TCID_50_ estimation. Additional 500 μL samples were taken and stored frozen (-80° C) at days 3, 10, 17, 24 and 31 for future use. From these, samples from day 3 and day 10 were thawed and used during week 2 and 3, respectively, to enhance the temporal resolution of the TCID_50_ decline. The viral decline was examined by dividing the virus titres (N_t_) by the initial viral concentration (N_0_) for each time point and then log transforming the quotients (N_t_ / N_0_). A statistical comparison between treatments was made using an ANOVA and a post-hoc Tukey test on the log transformed data after checking for homogeneity of variance using the Levene’s Test. All these analyses were performed in the free software environment of statistical computing R using the ‘car’ and ‘multcomp” packages [27, 28].

## 3. Results

The first pilot studies primarily show the efficacy of the chosen methodology. No cytotoxicity was observed in the negative control treatments, while good correspondence was observed between the T_0_ virus titres of all treatments (Tables 1 and 2). Based on our past experiences using these strains of PRCV and ST cells, we consider the apparent differences in T_0_-titres of all treatments non-significant as they are in line with the usual variability observed during TCID_50_ assays. Here, this technical variability can be observed by comparing the T_0_-measurements of the 15° C and 20° C samples, which are biological replicates at that point as they have not been incubated yet. We found an average difference of 0.30 and 0.40 log(TCID_50_/mL)–which is an average standard deviation of 0.21 and 0.28 log(TCID_50_/mL)–between the T_0_- titres of the 15° C and 20° C samples of the same media in the first and second experiment, respectively. Due to the lack of biological replicates and therefore the possibility for statistical testing, any perceived difference over time or between treatments should considerably exceed this variability–ideally by an order of magnitude–to be relevant. With this in mind, we were not able to observe any changes over time and, hence, did not detect significant declines in virus titres at neither temperature within the first 3 days.

**Table 1:** The short-term stability of PRCV in natural and artificial salt- and freshwater media.

	Log(TCID_50_/mL)				Log(TCID_50_/mL)		
**15°C**	0 h	2 h	4 h	**20°C**	0 h	2 h	4 h
PBS	6.30	5.80	6.30		6.30	5.80	6.30
Seawater	6.30	5.63	5.96		5.80	5.96	5.63
Artificial seawater	5.53	5.80	5.80		5.80	5.63	6.07
**15°C**	0 h	2 h	4 h	**20°C**	0 h	2 h	4 h
PBS	5.80	5.96	5.96		6.30	5.96	6.30
River water	6.30	5.96	5.80		5.80	5.30	5.80
Mineral water	5.80	5.80	5.96		5.80	5.80	5.80

PBS was used as a positive control. Swine testicle cell cultures were used to determine the virus titres after 0, 2 and 4 hours of incubation at 15°C or 20°C. The values shown below are the log of the Tissue Culture Infectious Dose 50%/mL (TCID_50_%/mL). One assay was performed per treatment.

**Table 2:** The 3-day stability of PRCV in natural and artificial salt- and freshwater media.

	Log(TCID_50_/mL)				Log(TCID_50_/mL)		
15°C	0 d	1 d	3 d	20°C	0 d	1 d	3 d
PBS	6.63	6.63	6.80		6.96	5.80	6.63
Seawater	5.96	6.07	5.96		6.80	5.96	6.63
Artificial seawater	5.96	6.13	6.30		6.30	5.96	5.80
River water	6.63	6.30	5.63		6.63	6.30	5.96
Mineral water	6.3	6.30	6.30		6.80	6.63	5.80

PBS was used as a positive control. Swine testicle cell cultures were used to determine the virus titres after 0, 1 and 3 days of incubation at 15°C or 20°C. The values shown below are the log of the Tissue Culture Infectious Dose 50%/mL (TCID_50_). One assay was performed per treatment.

No short-term inactivation of PRCV was found during the third pilot study either (Table 3). The PRCV titres were the same after 6 hours of incubation for both the PBS control treatment and the unfiltered natural seawater treatment. In the concentrated samples, we observed a 32% and 46% reduction in TCID_50_ estimates for the 10x and 100x higher particle loaded media, respectively, which may not be significant given the aforementioned technical variability of our test. Unlike the first experiments, there are no replicates at T_0_ to compare. Yet, as the first and second experiment employed the same number of wells per dilution during the TCID_50_ assay as exp. 3, at least similar technical variability is expected. An average standard error of the mean of 0.17 log(TCID_50_/mL) was reported for the first experiments. When applied here, this means that up to 30% reduction in viral loads may fall within one standard error of the mean TCID_50_ estimate.

**Table 3:** PRCV was incubated in seawater media with 1x, 10x, or 100x the natural concentration of suspended solids (>100 kDa).

	Log(TCID_50_/mL)	
20°C	0h	6h
PBS	6.80	6.80
Seawater	6.96	6.96
10x Concentrate	6.80	6.63
100x Concentrate	6.80	6.53

PBS was used as a positive control. Swine testis cells were used to determine virus titres after 6 hours of incubation at 20°C. The values shown are the log of the Tissue Culture Infectious Dose 50%/mL (TCID_50_). One assay was performed per treatment.

**Fig 1:**
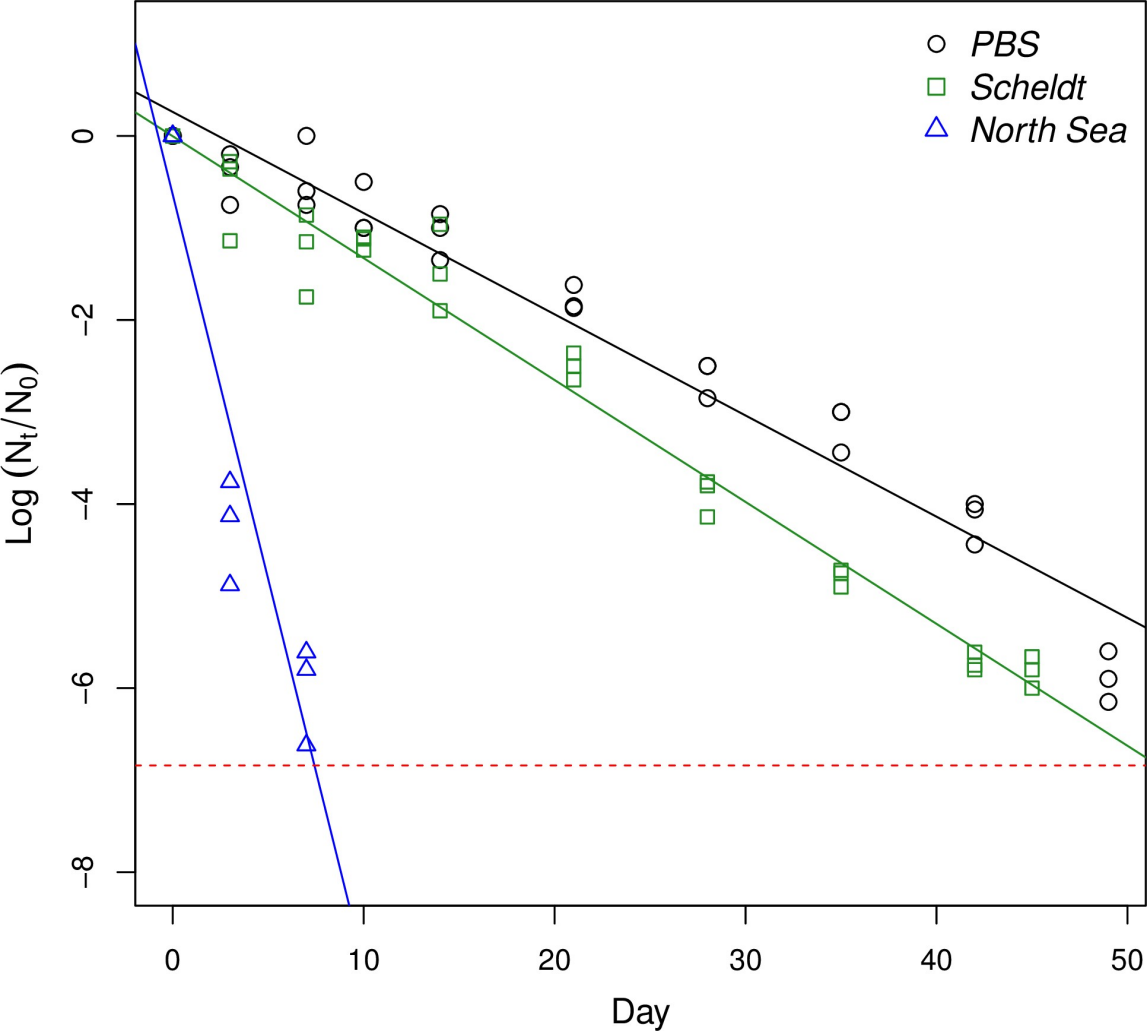
Long-term viral inactivation of PRCV, kept in the dark at 20°C in PBS, river water (Scheldt) and natural seawater (North Sea). Values shown represent log_10_ reductions in viral load at each time point. Dashed line represents the limit of detection of our test.

Viral inactivation was observed during the last, long-term experiment in all three treatments. The PRCV titres decreased faster in both the filtered river water and the filtered seawater relative to the PBS control treatment (Fig 1; Tukey HSD p < 0.01 and p < 0.001, respectively). During our experiment, the virulence of PRCV, as measured by the TCID_50_ assay, decreased by 99% and 99.9% after 19.6 days (T_99_) and 29.4 days (T_99.9_), respectively, when kept in PBS at 20°C in the dark. By contrast, 99% of the virus was inactivated after staying 15.4 days in filtered water from the Scheldt (T_99_) and as little as 2.2 days in filtered seawater (T_99_, North Sea). Using the same regression analyses, the inactivation times for 50%, 90%, 99%, 99.9% and 99.99% of PRCV were also predicted for all three treatments (Table 4).

**Table 4:** Survival of PRCV in PBS, filtered river water and filtered seawater.

	PRCV strain 91V44 Survival (in days)				
20°C	T_50_	T_90_	T_99_	T_99.9_	T_99.99_
PBS	3.0	9.8	19.6	29.4	39.2
Scheldt river	2.8	8.0	15.4	22.8	30.2
North Sea	0.25	1.1	2.2	3.4	4.6

The slope of the linear regression was used to estimate the time, in days, for virus titres to decrease by 50% (T_50_), 90% (T_90_), 99% (T_99_), 99.9% (T_99.9_) and 99.99% (T_99.99_) when kept in the dark at 20°C.

## 4. Discussion

Waterborne transmission of viruses has long been recognized as a serious risk for human health. Some non-enveloped enteric viruses like *Norovirus* are notoriously able to survive the transit from wastewater to rivers, the ocean and then back to humans via seafood [21, 29, 30]. Faecal-oral transmission is less common in enveloped viruses, which seem more susceptible to inactivation in aquatic environments and, hence, underrepresented in waterborne virus research [23]. Some enveloped viruses do survive for prolonged periods in wastewater and fresh surface waters. Crucially, two high-profile groups of pathogens belong to this group of resilient enveloped viruses, namely the avian influenza viruses and coronaviruses [23]. Three infamous human coronaviruses (HCoV), SARS-CoV, MERS-CoV, and now SARS-CoV-2, can be found in stool and wastewaters, sparking concerns about the potential faecal-oral transmission of these viruses in areas with poor sanitation [22, 31]. Since viruses cannot replicate outside of their hosts, the risk of waterborne transmission depends on their ability to remain viable in aquatic environments. The limited information available suggests that CoVs are generally more unstable in the environment than non-enveloped viruses, but more data on their persistence in various matrices are needed [22].

The persistence of CoVs in aquatic environments is mostly studied using surrogate animal viruses like murine hepatitis virus MHV [32, 33], feline infectious peritonitis virus FIPV [34], and porcine transmissible gastroenteritis virus TGEV [32, 35], but several human CoVs have also been used. These include Human CoV strain OC43 [36], Human CoV strain 229E [34, 36, 37], SARS-CoV [37–39], MERS-CoV [40], and more recently SARS-CoV-2 [41, 42]. The stability of CoVs has been studied in various aquatic media, ranging from reagent-grade water and culture growth media to wastewater and milk. To our knowledge, only one study to date has investigated the stability of CoVs in seawater [42]. Here, we used the porcine respiratory coronavirus (PRCV), a CoV that has not been used yet as a surrogate CoV in these types of experiments, to study the persistence of CoVs in natural fresh- and saltwater matrices.

Table 4 and Fig 1 show the survival of PRCV in PBS, filtered freshwater from the river Scheldt and filtered seawater from the North Sea. PRCV remained virulent for the entire duration of the experiment (49 days) when kept at 20°C in PBS, showing only a slow decline with a half-life of approximately 3 days in the PBS control treatment. By comparison, Sizun et al. (2000) found that the half-life of HCoV-229E and HCoV-OC43 in PBS is around 5 days and 3 days, respectively, at a higher temperature of 37°C [36]. While we did not directly observe a temperature effect due to the length and sensitivity of the pilot experiment, the temperature response of PRCV is expected to be similar to the trends observed for other CoVs. As temperature is positively correlated with inactivation rates of CoVs [43], these results indicate that PRCV is more readily inactivated than HCoV-229E and HCoV-OC43, which are expected to have even longer half-lives at 20°C. By contrast, SARS-CoV was shown to be more stable than HCoV-229E in cell culture supernatants at room temperature [37], which then suggests that PRCV may be less resilient than SARS-CoV as well. Wang et al. (2005) have found that SARS-CoV remains detectable after 14 days in PBS at 20°C, which was the full duration of their experiment [38]. Due to the limited number of studies available, these are the only direct comparisons that can be made between the stability of PRCV and other CoVs suspended in buffers or culture media. Looking at freshwater, the current study reports that PRCV titres declined by 99.9% after 15.4 days in filtered water from the river Scheldt at 20°C. Similarly, Casanova et al. (2009) found that TGEV and MHV are reduced by 99.9% after 13 days and 10 days in natural lake water at 25°C [32]. Recently, Sala-Comerera et al. (2021) reported that SARS-CoV-2 is reduced by 90% after 2.3 days in filtered river water at 20°C [42], which is considerably faster than PRCV. In tap water, SARS-CoV is known to persist for two days at 20°C [37]. Another recent report by Bivins et al. (2020) found similar rates of inactivation for SARS-CoV-2 in tap water [41]. These rates are significantly faster than the 12.1 and 12.5 days found at 23°C for HCoV-229E and FIPV [34].

This study is the first to measure the environmental stability of PRCV in seawater. During the final experiment, PRCV titres decreased by 90% and 99% after 1.1 and 2.2 days, respectively, which is identical to the T_90_ which was recently reported for SARS-CoV-2 in seawater at 20°C [42]. This rapid decline was, however, not observed during the preceding experiments. Laude (1981) has previously suggested that viral inactivation is greater at alkaline than at neutral pH [35], but this mechanism alone cannot be responsible for the mixed results, as neither the artificial seawater nor filtered North Sea water differed from the PBS positive control during the first two experiments. Despite the short exposure times and the limited sensitivity of the first tests, a trend should have been observed if either pH or salinity would cause a rapid decline in viral titres. Ye et al. (2016) reported significant sorption of enveloped viruses like MHV to suspended solids [33]. During the third experiment, a trend was indeed observed whereby PRCV titres decreased with increasing concentrations of suspended solids. Sorption to particles was, however, excluded during all other experiments through filtration and hence does not provide an explanation for the variable results. The presence of predatory microorganisms like protozoa and non-host macroorganisms that also reduce viral abundance within the natural ecosystem [44–46] was similarly excluded from our experimental setup. Noble and Fuhrman (1997) found that filtration reduces viral decay rates in coastal seawater by 20% on average [18]. They, and others, go on to suggest that heat-labile dissolved organic matter (>30 kDa), extracellular proteases and nucleases from microorganisms are key contributors—second only to UV-C degradation—of viral decline in coastal ecosystems [17, 18, 30, 47]. Heat-labile, high-MW molecules and colloids between 30 kDa and 0.2 μm were found to be responsible for about 20 to 25% degradation of marine phages [18]. A large fraction of organic matter was most likely not retained by our ultrafiltration setup, given that more than 90% of the protein-like components in seawater are smaller than 3 kDa [48], and hence did not contribute to the potential response to increased particle loads. Differences in larger-sized organic matter in our seawater can, however, explain the difference in response between the experiments.

A significantly longer filtration time was needed to prepare our seawater for the last experiment, which is usually caused by blooms of *Phaeocystis* spp. in the Belgian coastal zone. Shear stress during the vacuum filtration may have damaged or ruptured some of the phytoplankton, artificially increasing both the high-MW and low-MW fraction of organic matter available, which in turn could increase the viral degradation rates during our tests. Similarly, tangential flow ultrafiltration may have released some intracellular organic matter during experiment 3, which may have contributed to the observed trend. This interference should, however, be minimal as the Vivaflow ultrafiltration cassettes were used in conjunction with a peristaltic pump, which was reported to have minimal effect on the microphytoplankton and picoplankton [49, 50]. Both the natural availability of organic matter and the amount of interference caused by filtration-induced cell rupture is dependent on the natural primary production and, hence, will vary in space and time. Future experiments should consider including measurements of organic matter concentrations to have more control over this source of variability. Another thing to keep in mind is that UV-induced viral decay was excluded from our study by working in the dark. While a similar approach was used to assess the survival of SARS-CoV-2 in seawater [42], it should be clear that these experimental setups are likely to underestimate viral decay under natural conditions. In addition to UV-C induced viral decay, these experiments exclude food web interactions and the kinetics of sorption to suspended solids that are known to affect viral concentrations. With that in mind, our results support the consensus that a significant accumulation of terrestrial CoVs in the coastal environment is unlikely. Attempts to find infectious SARS-CoV-2 in seawater have so far failed [51, 52]. More research is, however, still needed to understand the environmental fate of CoVs. Studies have shown that significant spatiotemporal variability in marine viral decay is likely due to fluctuating environmental conditions as well as species-specificity. New studies should focus on the structural characteristics of CoVs to better understand the mechanisms that lead up to their environmental demise [22, 23].

## 5. Conclusions

This study used the Porcine Respiratory Coronavirus (PRCV) to examine the environmental fate of coronaviruses (CoV) in aquatic environments. The results are in good agreement with previous studies on other surrogate and human CoVs. CoVs are persistent in freshwater environments, a trait that can and has been exploited for the monitoring of epidemics such as COVID-19 through wastewater management. A rapid decline in viral infectivity is expected in coastal systems, especially in a highly productive area like the Southern North Sea. Significant spatiotemporal variation should, however, be expected. To date, we lack a fundamental understanding of all the mechanisms at play. Coupled to species-specific variation in environmental resilience of CoVs, it becomes clear that more research in this field is still needed.

## Supporting information

S1 Data (CSV)
